# Active Glucose Transport 2020 and Beyond

**DOI:** 10.1093/function/zqaa047

**Published:** 2020-12-21

**Authors:** Ernest M Wright, Donald D F Loo

**Affiliations:** Physiology Department, David Geffen School of Medicine at UCLA, Los Angeles, CA, USA; Physiology Department, David Geffen School of Medicine at UCLA, Los Angeles, CA, USA

As a concept, active transport emerged from early studies of intestinal glucose absorption and renal glucose reabsorption. Transport requires energy and is blocked by the natural glucoside phlorizin. Bob Crane proposed in 1960 that the energy comes directly from the Na^+^ gradient across the membrane.^1^ The sodium–glucose cotransport hypothesis has been tested, confirmed, and extended to the active transport of molecules into cells throughout the body, eg, neurotransmitter uptake into neurons. The intestinal glucose cotransporter (SGLT1) was the first to be identified, cloned, and studied in heterologous expression systems such as Xenopus oocytes and cultured cells.^1^ SGLT1 is the founding member of the SLC5 human gene family^1^ and the large APC superfamily found throughout all life forms (www.tcdb.org).

Extensive functional, biochemical, biophysical, and molecular genetic studies have resulted in kinetic and structural models for the mechanism of active glucose transport^1^^,^^2^^,^^3^ (Figure 1). SGLT1 is an integral membrane protein with a core of 10 transmembrane helices arranged in an inverted repeat in common with the APC superfamily members. The glucose-binding site is in the middle of the protein and access is via aqueous channels with outer and inner gates. Functionally, the driving cation, Na^+^, binds to the apoprotein to open the external channel and gate to allow glucose to bind (Figure 1A).^7^^,^^8^ The external gate closes, the water channel collapses, and sugar-binding triggers the opening of the inner gate and inner channel, and the coupled transport of Na^+^ and glucose to the cytoplasm (Figure 1B). The transporter is reversible, where the rate and direction of transport are simply governed by the Na^+^ and glucose concentration on each side of the membrane, and the membrane potential. The voltage dependence of transport is due to the apparent charge of the protein (*z* = 1), and fast SGLT1 capacitive charge enables determination of protein conformation changes in real time.^7^^,^^8^ Water permeates through the glucose transport pathway.^2^ Phlorizin and SGLT2 drugs bind to the external face of the transporter with the glucose moiety in the glucose-binding site and the aglycone in the external channel.^4^ Unresolved questions include the identity of the voltage sensor, and the nature of the power stroke of cotransport.

**Figure 1.**
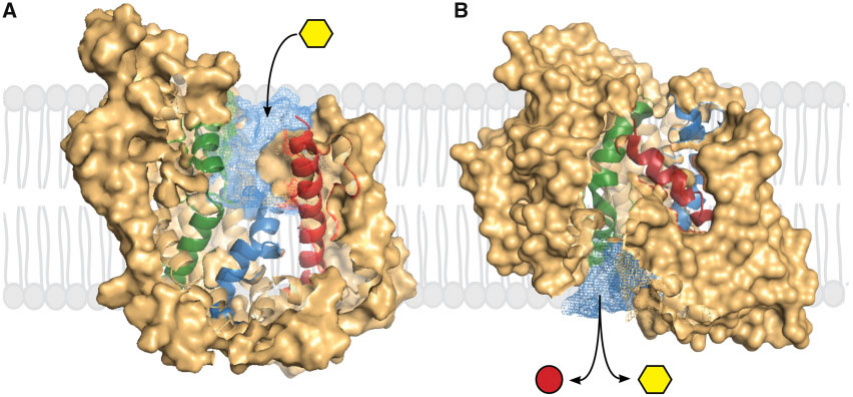
Na^+^/Glucose Transport Through SGLTs. The protein is shown in two conformations (**A**) outward-facing open and (**B**) inward-facing open.^4^^,^^5^ The cross-sectional views show the external aqueous channel leading to the glucose binding site, and the internal channel leading from the glucose-binding site to the cytoplasm. The external channel opens in the presence of external Na^+^ and closes after external sugar-binding when the internal channel opens to release glucose and Na^+^ to the cytoplasm.^6^ The reciprocal opening and closing of the channels are brought about by local motions of transmembrane helices. The closure of the external channel after glucose binding is brought about by the inward movements of the external ends of TM1 (green), TM6 (blue), and TM10 (red), while the opening of the internal channel is caused by the outward movements of the internal ends of TM1 and TM5. SGLT1 requires two Na^+^ ions for cotransport, while SGLT2 only requires one.

SGLT1 mutations cause glucose–galactose malabsorption, and SGLT2 mutations cause familiar renal glycosuria.^1^^,^^3^ SGLT2 is uniquely expressed in the renal proximal tubule, and the pharmaceutical industry has successfully produced phlorizin analogs to treat T2DM (Type 2 diabetes).^2^^,^^3^ Interestingly, the SGLT2 drugs promote weight loss, and significantly reduce death and hospitalization due to left heart failure.^9^

Future SGLT research will reveal higher resolution structures in multiple conformational states, through new IT advances such AlphaFold by Google company DeepMind, cryo-EM of transporters embedded in liposomes.^10^ New structures together with the results on the dynamics of conformational changes in real time, using perturbation methods such as voltage–clamp fluorometry,^8^ will provide new insights into transport mechanism. All will be consolidated using new tools of molecular dynamics.^4^^,^^6^ On a clinical note, we expect further progress on SGLT inhibitors to treat T2DM, T1DM, and cancer, advances in understanding the exciting effects of SGLT2 in treating heart failure patients, and the role of SGLTs in T-lymphocytes, and early pregnancy.^2^

## Acknowledgements

We are grateful to our colleagues and collaborators who have contributes so much to this SGLT story. We thank Dr Jeff Abramson for his assistance with Figure 1.

## Funding

Supported by grants from the National Institutes of Health.

## Conflict of Interest Statement

None.

## References

[1] Wright EM , Loo DD, Hirayama BA. Biology of human sodium glucose transporters. Physiol Rev 2011;91:733–794. 21527736 10.1152/physrev.00055.2009

[2] Wright EM , Ghezzi C, Loo DDF. Novel and unexpected functions of SGLTs. Physiology 2017;32:435–443. 29021363 10.1152/physiol.00021.2017 PMC5817162

[3] Ghezzi C , Loo DDF, Wright EM. Physiology of renal glucose handling via SGLT1, SGLT2 and GLUT2. Diabetologia 2018;61:2087–2097. 30132032 10.1007/s00125-018-4656-5 PMC6133168

[4] Bisignano P , Ghezzi C, Jo H, et al Inhibitor binding mode and allosteric regulation of Na^+^-glucose symporters. Nat Commun 2018;7:5245. 10.1038/s41467-018-07700-1 PMC6286348 30532032

[5] Paz A , Claxton DP, Kumar JP, et al Conformational transitions of the sodium-dependent sugar transporter vSGLT. Proc Natl Acad Sci USA 2018;115:E2742–E2751. 29507231 10.1073/pnas.1718451115 PMC5866573

[6] Adelman JL , Ghezzi C, Bisignano P, et al Stochastic steps in secondary active transport. Proc Natl Acad Sci USA 2016;113:E3960–E3966. 27325773 10.1073/pnas.1525378113 PMC4941443

[7] Loo DD , Jiang X, Gorraitz E, Hirayama BA, Wright EM. Functional identification and characterization of sodium binding sites in Na symporters. Proc Natl Acad Sci USA 2013;110:E4557–E4566. 24191006 10.1073/pnas.1319218110 PMC3839715

[8] Gorraitz E , Hirayama BA, Paz A, Wright EM, Loo DDF. Active site voltage clamp fluorometry of the sodium glucose cotransporter hSGLT1. Proc Natl Acad Sci USA 2017;114:E9980–E9988. 29087341 10.1073/pnas.1713899114 PMC5699082

[9] Cowie MR , Fisher M. SGLT2 inhibitors: mechanisms of cardiovascular benefit beyond glycaemic control. Nat Rev Cardiol 2020;17:761–772. 32665641 10.1038/s41569-020-0406-8

[10] Yao X , Fan X, Yan N. Cryo-EM analysis of a membrane protein embedded in the liposome. Proc Natl Acad Sci USA 2020;117:18497–18503. 32680969 10.1073/pnas.2009385117 PMC7414195

